# Genetic diversity is positively associated with fine-scale momentary abundance of an invasive ant

**DOI:** 10.1002/ece3.313

**Published:** 2012-07-24

**Authors:** Monica A M Gruber, Benjamin D Hoffmann, Peter A Ritchie, Philip J Lester

**Affiliations:** 1 School of Biological Sciences, Victoria University of WellingtonPO Box 600, Wellington, 6140, New Zealand; 2 CSIRO Ecosystem Sciences, Tropical Ecosystems Research CentrePMB 44, Winnellie, Northern Territory, 0822, Australia

**Keywords:** Australia, genetic paradox, invasive species, social insects, unicoloniality

## Abstract

Many introduced species become invasive despite genetic bottlenecks that should, in theory, decrease the chances of invasion success. By contrast, population genetic bottlenecks have been hypothesized to increase the invasion success of unicolonial ants by increasing the genetic similarity between descendent populations, thus promoting co-operation. We investigated these alternate hypotheses in the unicolonial yellow crazy ant, *Anoplolepis gracilipes*, which has invaded Arnhem Land in Australia's Northern Territory. We used momentary abundance as a surrogate measure of invasion success, and investigated the relationship between *A. gracilipes* genetic diversity and its abundance, and the effect of its abundance on species diversity and community structure. We also investigated whether selected habitat characteristics contributed to differences in *A. gracilipes* abundance, for which we found no evidence. Our results revealed a significant positive association between *A. gracilipes* genetic diversity and abundance. Invaded communities were less diverse and differed in structure from uninvaded communities, and these effects were stronger as *A. gracilipes* abundance increased. These results contradict the hypothesis that genetic bottlenecks may promote unicoloniality. However, our *A. gracilipes* study population has diverged since its introduction, which may have obscured evidence of the bottleneck that would likely have occurred on arrival. The relative importance of genetic diversity to invasion success may be context dependent, and the role of genetic diversity may be more obvious in the absence of highly favorable novel ecological conditions.

## Introduction

The success of invasive species is a genetic paradox because the loss of genetic diversity that invading populations typically experience should, in theory, limit the chances of invasion success (Allendorf and Lundquist 2003). The genetic bottlenecks that are typically experienced by small founding populations often result in reduced genetic variation relative to the parent population (Sakai et al. 2001). In the short term, small introduced populations are thus susceptible to inbreeding and strong genetic drift, which may further erode genetic variation. In the longer term, a lack of genetic variation may impede the potential for adaptive evolution. This apparent genetic paradox may not be evident if invading populations have higher genetic diversity than source populations due to high propagule pressure (Kolbe et al. 2004; Roman and Darling 2007), the genetic diversity measured in commonly used molecular markers such as microsatellites does not reflect the total diversity in the genome (Väli et al. 2008), or if genetic variation is unimportant, as is observed in clonally reproducing species (Baker 1995; Dybdahl and Drown 2011).

In some instances colonization success may be enhanced by a reduction in genetic diversity. In social and colonial animals, for example, high genetic similarity due to a small number of founders in the introduced population may result in increased co-operation, thus enhancing colonization success. This should theoretically be the case for ants, where kin selection promotes altruism among closely related individuals (Hamilton 1964), and co-operation facilitates ecological success (Hölldobler and Wilson 1990). A further paradox, however, is that many invasive ants are unicolonial species that can co-operate despite relatively low relatedness reported within the colony (reviewed by Helanterä et al. 2009). According to the assumptions of kin-selection theory, the evolutionary lifespan of these lineages may thus be limited (Helanterä et al. 2009). Researchers have suggested, however, that higher genetic similarity owing to population bottlenecks during the introduction event may have promoted unicoloniality, and thus invasion success, of the well-studied Argentine ant *Linepithema humile* (Suarez et al. 1999; Tsutsui et al. 2000). This bottleneck hypothesis has been disputed by others, however (Vogel et al. 2010), and an alternative hypothesis has been suggested that “genetic cleansing” at recognition alleles may promote unicoloniality in *L. humile* (Giraud et al. 2002). However, genetic bottlenecks may also aid in invasion success through the purging of deleterious alleles (Schmid-Hempel et al. 2007). As the bottleneck hypothesis specifically refers to differences between the native and introduced ranges (Suarez et al. 1999; Tsutsui et al. 2000), the putative benefits of a loss of genetic diversity may or may not persist subsequent to the initial bottleneck.

Invasion success can be difficult to define and measure. However, the effects of invasive ant species on the recipient community are density dependent (e.g., Ross et al. 1996; O'Dowd et al. 2003; Le Breton et al. 2005; Krushelnycky and Gillespie 2008; Lester et al. 2009). The high abundance attained by invasive ants strengthens their competitive ability relative to native ants and furthers their ecological success through numerical dominance of resources (e.g., Holway 1999; Human and Gordon 1999; Morrison 2000; Rowles and O'Dowd 2007; Sagata and Lester 2009). In most organisms the census population size (*N*_c_) and effective population size (*N*_e_) are coupled, and genetic diversity typically increases with increasing population size. In ant species, however, worker abundance does not reflect effective population size, as reproduction involves relatively few individuals, and these are not workers (Wilson 1963). Thus, as effective population size and worker abundance are independent in ants, worker abundance, along with the alteration of community structure in the invaded community (e.g., Sanders et al. 2003) are proxies by which short-term invasion success may be assessed. Here, we define short-term invasion success as the ability for a species to reach sufficient momentary abundance to result in a reduction in species diversity and a change in structure of the community into which they have been introduced.

*Anoplolepis gracilipes* is one of the most widespread, abundant, and damaging invasive ants (Holway et al. 2002). It can occur at very high densities (>2000 ants/m^2^) and can be a driver of substantial ecosystem change (O'Dowd et al. 1999, 2003; Abbott 2006). In our study population in Arnhem Land in the Northern Territory of Australia, these invasive ants show patterns of abundance that differ spatially. Unlike *A. gracilipes* invasions in Samoa (Savage et al. 2009) and on Christmas Island (O'Dowd et al. 2003), there appear to be no obvious single ecological mechanisms promoting differences in abundance, such as mutualisms with Homoptera, nor are there clear associations between the abundance of this ant and anthropogenic disturbance (Hoffmann and Saul 2010). We therefore hypothesized that there could be an association between genetic diversity and the variation in abundance of *A. gracilipes* in Arnhem Land.

Although the genetic structure and behavior of *A. gracilipes* in Arnhem Land is consistent with a single population, the population is apparently in the process of divergence (Gruber et al. 2012), hypothesized to be driven by genetic drift (Drescher et al. 2010). Thus, the variable, but spatially discrete occurrences of *A. gracilipes* in Arnhem Land resemble a mosaic of distinct nest clusters or “meta-colony” (*sensu* Heller et al. 2008). In this study we use “population” to refer to the entire distribution of *A. gracilipes* in Arnhem Land, and “nest clusters” to refer to individual localized occurrences of the ant.

**Figure 1:**
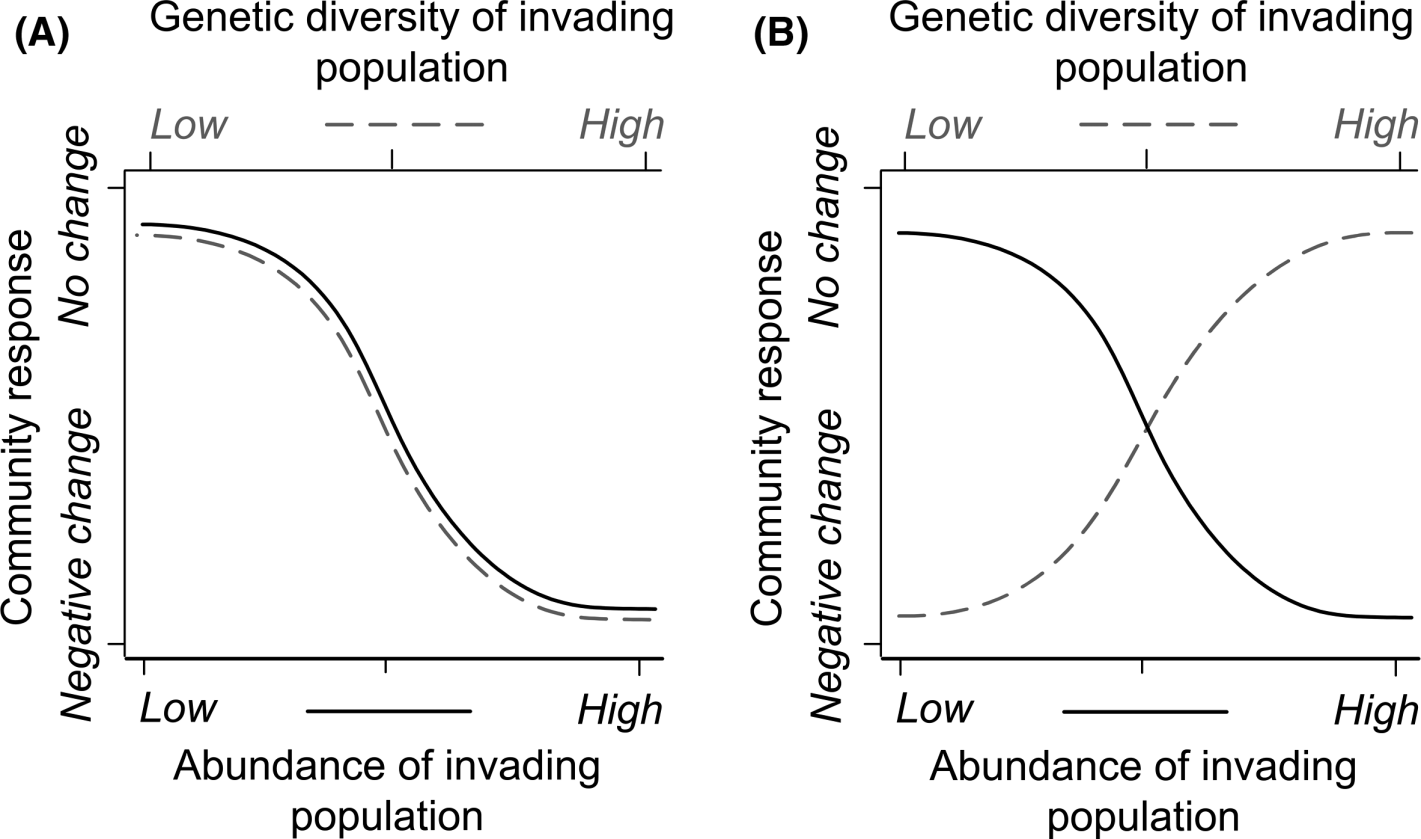
Our hypothesized relationship between the genetic diversity (e.g., allelic richness or genotypic richness) and abundance of the invading population, and relative influence on invaded communities under scenarios of: (A) a positive relationship between genetic diversity and invasion success and (B) a negative relationship between genetic diversity and invasion success.

The aim of this study is to explore the relationships between genetic diversity, abundance, and ecological success. The study of Gruber et al. (2012) investigated population genetic differentiation (i.e. beta diversity), whereas in this study we extend the analysis to investigate genetic diversity at the local scale at which ants are likely to interact (i.e. alpha diversity). If higher genetic diversity is associated with higher momentary abundance (or short-term invasion success), higher genetic diversity and greater abundance would be co-observed, and would be associated with negative effects on the invaded community (Fig. 1A). Conversely, if lower genetic diversity is associated with invasion success, lower genetic diversity would be correlated with greater abundance and with negative effects on the invaded community (Fig. 1B). To test these alternate hypotheses we address three specific questions: (1) Is there an association between *A. gracilipes* genetic diversity and abundance? (2) Are there differences in native ant species diversity and community structure between invaded and uninvaded communities? (3) Is there an association between *A. gracilipes* abundance and native ant species diversity in the invaded community? Furthermore, to evaluate an effect of environment we asked: (4) Are habitat characteristics associated with variation in *A. gracilipes* abundance?

## Materials and methods

### Study area

**Figure 2:**
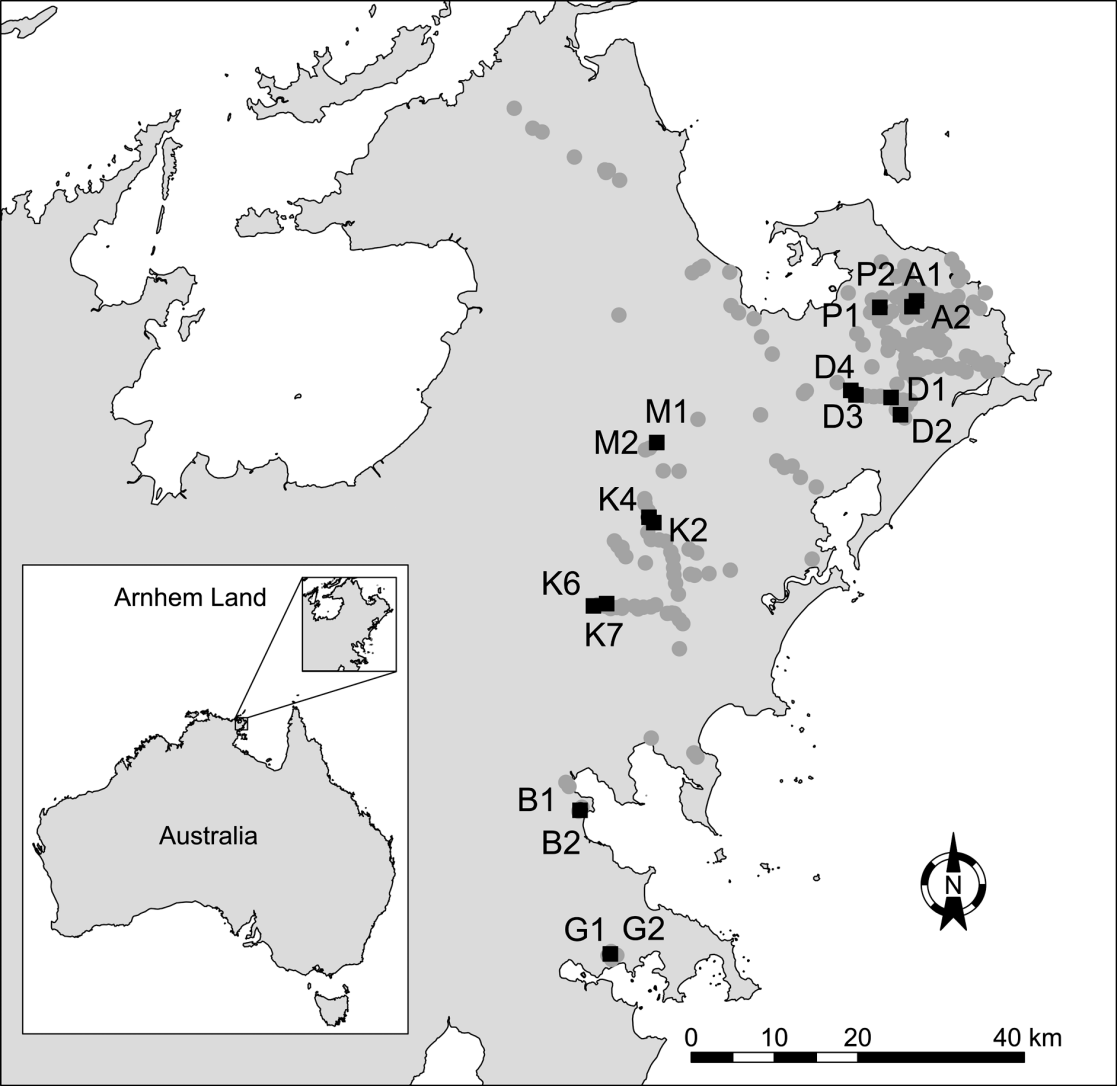
*Anoplolepis gracilipes* distribution at the time of sampling (gray circles) and sampling sites (black squares with site codes) in Arnhem Land in the Northern Territory, Australia.

Arnhem Land is located in the monsoonal tropics of Australia's Northern Territory (Fig. 2). The region experiences daytime high temperatures ranging between 17 and 33°C, and seasonal rainfall (December–July) of approximately 1200 mm. The vegetation is primarily fire-prone savanna woodland. Although it is not known when *A. gracilipes* arrived in the region, it was first detected in 1982 (Majer 1984). In 2009 the ant was patchily distributed throughout 16,000 km^2^ in Arnhem Land (Fig. 2), mainly in undisturbed and ecologically intact sites. Genetic and behavioral analyses suggest this population stemmed from a single source (Gruber et al. 2012). However, the genetic structure and intra-specific behavior of the population is heterogeneous, which suggests the population is in the process of divergence (Gruber et al. 2012).

### Ecological surveys

Nine study sites were selected for an ecological survey undertaken in July 2009 (Fig. 2), which intersect with the sites sampled in Gruber et al. (2012). The differences were the addition of plots D1, M1, K6, K7, and K2, additional genotyping, and ecological surveys in this study. We selected sites with similar habitat characteristics in a stratified random fashion. All sites had a dominant canopy of *Eucalyptus tetrodonta*, an understory primarily consisting of *Acacia* spp., grasses, and leaf litter, and similar drainage and topography. At each of the nine sites we selected two visually similar plots in areas invaded by *A. gracilipes*. Plots were spaced at least 100 m apart to ensure their independence. The largest *A. gracilipes* foraging distance observed in this region is 35 m, and within this distance ants freely move between nests (B. Hoffmann unpubl. data). The presence of at least one *A. gracilipes* nest was confirmed in invaded plots, and we paired these with nearby plots where the ant was absent.

At each plot we installed 16 pitfall traps (45 mm diameter) placed in a 4 × 4 m grid around an *A. gracilipes* nest. Traps were 2/3 filled with propylene glycol and left for 48 h. All ant species collected were counted and identified to species level and named where possible. Species that could not be named were assigned to species groups according to Andersen (2000). Voucher specimens for all species were retained at the Tropical Ecosystems Research Centre in Darwin or Victoria University of Wellington.

In addition to *A. gracilipes* counts in pitfall traps, to assess population densities we also measured the abundance of *A. gracilipes* at each plot based on forager activity, as described in Gruber et al. (2012). Briefly, this method uses a count of the number of ants crossing a laminated card in a 30 sec period (Green et al. 2004). At each plot we measured *A. gracilipes* activity at 11 stations spaced at 5 m intervals along three replicate 50 m transects spaced 10 m apart. Counts at all stations within a transect were summed, and the mean value of the three replicate transects was used as an index of relative abundance between plots. Our card counts ranged from 0 to 42, and were highly correlated with pitfall trap counts for each plot (Spearman's rank correlation *R*_s_ = 0.80, *S* = 195, *P* < 0.001), conducted in R v 2.13.1 (Ihaka and Gentleman 1996; R Development Core Team 2011). We used the pitfall trap data in all analyses as these data were collected at the same spatial scale as the genetic diversity and habitat data.

Native ant species were also assigned to the functional groups used in studies of Australian ant communities (Andersen 1995): Dominant Dolichoderinae (DD); Subordinate Camponotini (SC); Climate Specialists [sub-divided into Hot (HCS) and Tropical (TCS)]; Generalized Myrmicinae (GM); Opportunists (OPP); Cryptic Species (CS); and Specialist Predators (SP). In addition to *A. gracilipes* we found three other non-native ant species: *Monomorium floricola* (one ant in a single uninvaded plot), *Tetramorium simillimum* (62 ants in one invaded and five uninvaded plots), and *Paratrechina longicornis* (14 ants in two uninvaded plots). As these species are not known to have significant effects on savanna ant communities (Hoffmann and Saul 2010), we included their data with the native species.

We recorded a number of habitat attributes that we considered could contribute to differences in local ant community structure and abundances of *A. gracilipes*: availability of potential *A. gracilipes* nest sites (the proportion of the plot occupied by logs and tree basal area, as *A. gracilipes* often nest at the base of trees); canopy cover (measured with a spherical densiometer); the abundance of *Acacia* spp. (a count of the number of plants); and depth of leaf litter (a mean of four measurements). Denser canopy cover can facilitate extended foraging time in extreme heat. *Acacia* spp. offer a potential novel carbohydrate resource, which can enhance ant abundance (Davidson 1997 and references therein). Novel carbohydrate resources facilitate invasion of *A. gracilipes* on Christmas Island (O'Dowd et al. 2003) and Samoa (Savage et al. 2009). Finally, leaf litter depth is an approximate indicator of time since fire, with leaf litter deeper in unburnt areas (Cook 2003; Russell-Smith et al. 2009). Fire is a major driver of vegetation structure and composition in savannas, which in turn are major drivers of ant community composition. These habitat attributes are henceforth referred to as nest sites, canopy, *Acacia*, and litter.

### Molecular analyses

For molecular analyses, we haphazardly selected *A. gracilipes* workers from pitfall traps and nests in our study plots in July 2009 (Fig. 2). These samples were supplemented with additional ants collected from pitfall traps from the same plot when allele discovery curves did not flatten (Supplementary Fig. S1). Ants were stored in 95% ethanol at 4°C. We extracted genomic DNA using a modified Chelex protocol (Sepp et al. 1994). Individual workers were placed in microcentrifuge tubes, ground with sterile plastic pestles, and 150 μL of a 10% w/v Chelex-100 resin solution was added. The tubes were centrifuged briefly, boiled for 15 min, chilled on ice for 5 min, and centrifuged at 15,000 *g* for 15 min at 4°C. The supernatant containing DNA was stored at 4°C.

Microsatellite molecular markers were used to assess the genetic diversity of *A. gracilipes* at the 18 sampled plots. Workers were genotyped for seven microsatellite loci: *Ano1*, *Ano3*, *Ano4*, *Ano5*, *Ano7*, *Ano8*, and *Ano10* (Feldhaar et al. 2006), using the methods of Gruber et al. (2012). Fluorescent dyes used were FAM (*Ano4*, *Ano5*, and *Ano8*) and VIC (*Ano1*, *Ano3*, *Ano7*, and *Ano10*). Polymerase Chain Reactions were performed using the thermal cycling conditions specified by Feldhaar et al. (2006), with modification for M13 primers (Schuelke 2000). Amplified products were analyzed using the LIZ size standard on a 3730 Genetic Analyzer, and visualized and scored using GeneMapper v 3.7 (both Applied Biosystems, Foster City, California).

To determine whether sufficient worker ants were genotyped to represent allelic diversity, we generated allelic discovery curves using the “PopGenKit” package (Rioux Paquette 2011) in R, with jackknifing using 1000 replicates. A flattening of allele discovery curves with increasing sample size indicates that the samples genotyped are a fair representation of the allelic diversity in the population. Where possible, we genotyped additional ants for plots where discovery curves did not flatten (Supplementary Fig. S1). The majority of the 539 *A. gracilipes* workers genotyped were heterozygous at all loci, as is typical for this species (Drescher et al. 2007, 2010; Thomas et al. 2010; Gruber et al. 2012). Forty ants were homozygous at the *Ano8* locus, and one was homozygous at the *Ano5* locus. The undetermined reproductive mode of *A. gracilipes* (Drescher et al. 2007; Gruber 2012) and the existence of populations of *A. gracilipes* workers heterozygous at all loci (Thomas et al. 2010), indicates that homozygous loci could reflect allele dropout and thus be biologically inaccurate. We therefore conducted analyses with and without these worker genotypes as their presence could inflate genotypic diversity estimates. Although we found no major differences between the two datasets, we have chosen to report the analyses excluding these workers as they are more likely to be biologically accurate. These analyses were based on genotyping of 20–35 workers from each plot (mean ± SE = 27.67 ± 1.07, *n* = 498).

### Statistical analyses

For all statistical tests a significance level of α = 0.05 was used. For all analyses, effect sizes were interpreted as small (*R*^2^ ∼0.01, *R*_s_ ∼0.10), medium (*R*^2^ ∼0.09, *R*_s_ ∼0.30), or large (*R*^2^ ∼0.25, *R*_s_ ∼0.50), according to Cohen (1988).

#### Is there an association between *Anoplolepis gracilipes* genetic diversity and abundance?

Rarefied allelic richness was calculated in R using the “PopGenKit” package (Rioux Paquette 2011), with jackknifing of 1000 replicates. We estimated other genetic diversity parameters, including unbiased genotypic diversity (*R*_U_), Shannon entropy (*H′*), and Simpson's index of diversity (*D*) using Genclone v 2.0 (Arnaud-Haond and Belkhir 2007). We then derived Hill's numbers from these measures following the conventions of Jost (2006): ^*q*^*D*, where *q* = 1, 2, or 3 (^0^*D* = allelic richness [including only informative loci]; ^1^*D* = exp[Shannon's *H′*]; and ^2^*D* = 1/Simpson's *D*). These measures assign different weights to alleles (or species) depending on rarity, with the importance of rare alleles (or species) decreasing as *q* increases, and together provide a meaningful overview of diversity. We chose richness-based measures of diversity as these should be sensitive to the elimination of rare alleles by drift (Allendorf 1986). Genotypic diversity was chosen because the undetermined reproductive mode of *A. gracilipes* could involve clonality (Drescher et al. 2007; Heinze 2008; Gruber 2012), and genotypic diversity would provide an estimate of the number of reproductive clones. The relationships between diversity measures (^0^*D*, ^1^*D*, and ^2^*D*, and *R*_U_) and differences in abundance of *A. gracilipes* were analyzed using Spearman's rank correlation implemented in *R*. We visualized the relationships between variables by fitting smoothed lines with the loess function in *R* using a span of 0.9.

#### Are there differences in native ant species diversity and community structure between invaded and uninvaded communities?

Species diversity measures (richness, Shannon's *H′* and dominance/Simpson's *D*) were estimated using individual-based rarefaction (Hurlbert 1971) using 1000 permutations implemented in EcoSim V 7 (Gotelli and Entsminger 2007). The lowest number of native ants sampled in a plot (52 individuals) was the rarefied sample size. Hill's numbers were derived from these measures using the same convention as genetic diversity (^0^*D* = richness; ^1^*D* = exp[Shannon's *H′*]; and ^2^*D* = 1/Simpson's *D*). To test if these measures of species diversity differed between invaded and uninvaded plots, we used Wilcoxon rank sum tests (with Monte-Carlo resampling to estimate *P*-values), using the “coin” R package (Hothorn et al. 2008). We used the *Z*-score estimate to convert *Z* statistics to an *R*^2^ effect size using the formula *R*^2^ = *Z*^2^/*N* (Rosenthal 1991). Species accumulation curves were generated for invaded and uninvaded plots (Supplementary Fig. S2) using EstimateS v 8.2.0 (Colwell 2009).

To assess if ant community structure differed between invaded and uninvaded plots we used non-metric Multi-Dimensional Scaling (MDS) implemented in Primer V 6.1.11 (Clarke and Gorley 2006). We log-transformed data to even out the effects of rare and abundant species, and used the Bray–Curtis index as a similarity measure as recommended by Clarke and Warwick (2001). The MDS was run over 1000 iterations using Kruskal stress formula 1 and a minimum stress of 0.01. We tested for significant differences between invaded and uninvaded plots using analysis of similarity (ANOSIM). The ANOSIM analyses employed a two-way crossed design with treatment (invaded or uninvaded), and block (our nine sites) as factors and was run over 1000 permutations. We removed data for *A. gracilipes* before running the analyses. We used SIMPER to assess how individual species contributed to differences between invaded and uninvaded plots (Clarke and Warwick 2001).

#### Is there an association between *Anoplolepis gracilipes* abundance and native ant species diversity in the invaded community?

If higher abundance is associated with negative effects on the invaded community, we expected a decline in species diversity measures as abundance increased. To determine if species diversity measures (^0^*D*, ^1^*D*, and ^2^*D*) were correlated with differences in abundance of *A. gracilipes* we used Spearman's rank correlation in *R*. We visualized the relationships between variables by fitting smoothed lines with the loess function in *R* using a span of 0.9.

#### Are habitat characteristics associated with variation in *Anoplolepis gracilipes* abundance?

We used non-parametric multiple regression with generalized additive models and automatic spline smoothing implemented in the “mgcv” R package (Wood 2006) to test if the habitat attributes we measured contributed to differences in *A. gracilipes* abundance. We modeled *A. gracilipes* abundance as the dependent variable, site as a fixed factor, and litter, canopy, *Acacia*, nest sites as the independent variables. We ran models using all data, and data for invaded plots alone. As a single model could not be fit with all the model terms included, we used forward model selection, excluded terms that explained the least of the variation in the data, and selected the best models as those that explained most of the variation in the data. We visualized the relationships between environmental variables among plots using principal components analysis (PCA) in Primer V 6.1.11.

## Results

### Is there an association between *Anoplolepis gracilipes* genetic diversity and abundance?

**Figure 3:**
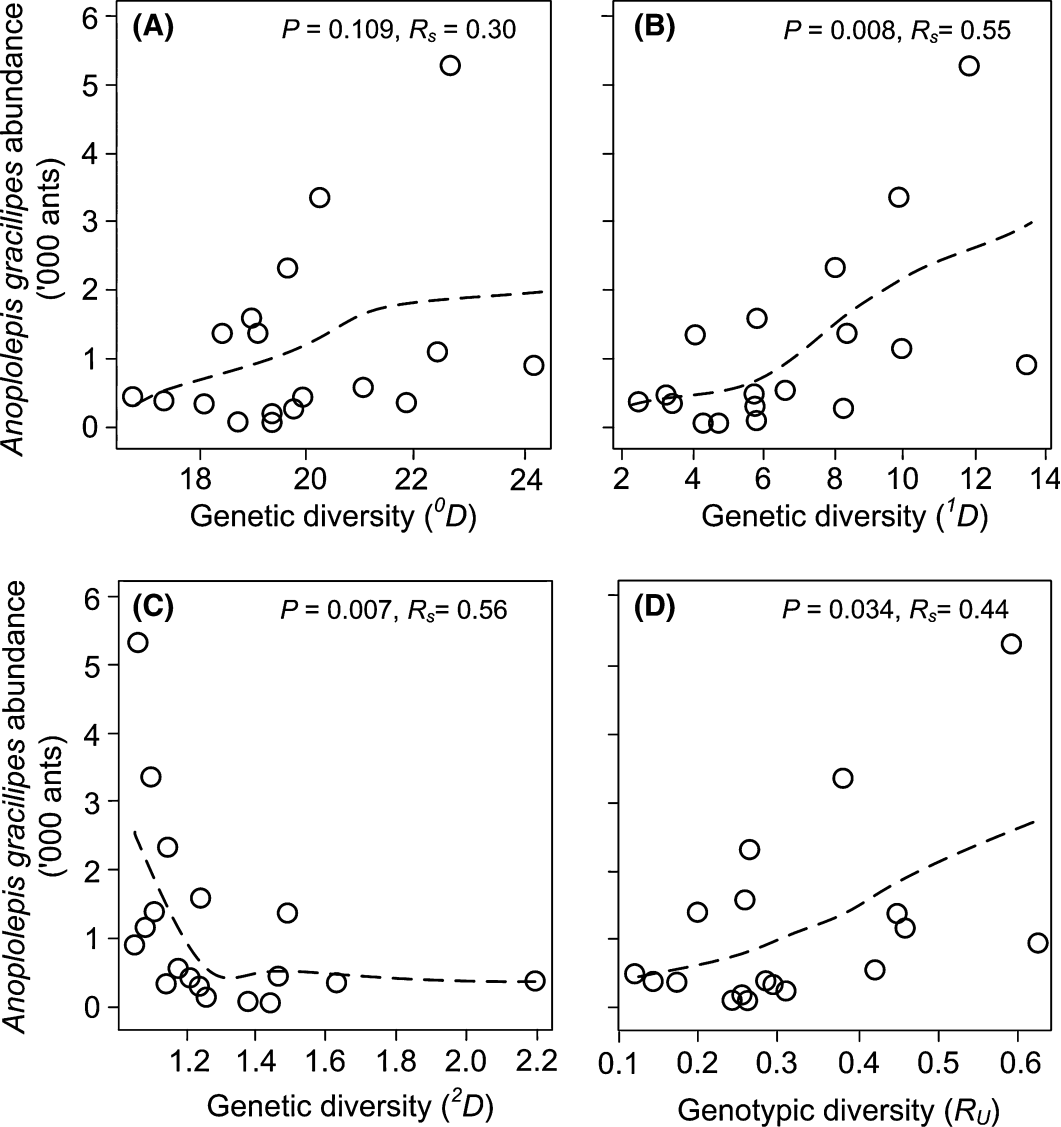
The relationship between *Anoplolepis gracilipes* abundance and genetic diversity measures: (A) ^0^*D* (allelic richness); (B) ^1^*D* (exp[Shannon's *H′*]); (C) ^2^*D* (1/Simpson's index *D*); and (D) genotypic diversity. Dashed lines indicate the smoothed spline line of best fit with a span of 0.9.

We found a significant correlation between most measures of genetic diversity and *A. gracilipes* abundance (Fig. 3). *Anoplolepis gracilipes* was the most abundant species in all invaded plots, but no single species was universally the most abundant in uninvaded plots. *Anoplolepis gracilipes* abundances in invaded plots ranged from 62 to 5288 ants per plot (mean ± SE: 1134 ± 334 ants), and from zero to 1016 ants per pitfall trap (mean ± SE: 71 ± 6 ants).

Allelic diversity measures changed markedly according to the value of *q*. When common and rare alleles were deemed equally important (^0^*D*) a positive association between diversity and abundance was found. This relationship strengthened when rare alleles were assigned somewhat less importance (^1^*D*). By contrast, when rare alleles were given much less importance than common alleles (^2^*D*) a negative association between diversity and abundance was evident (Fig. 3).

All genetic diversity measures were positively correlated with each other, with the exception of ^2^*D*, which was negatively correlated with the other measures (Table 1; Fig. 3). The abundance of *A. gracilipes* was also positively correlated with all genetic diversity measures with the exception of ^2^*D* (Fig. 3). The relationships between abundance and genetic diversity measures were not linear, and instead were best fit by lines that tended toward a sigmoidal or inverse exponential function. Although the relationship between *A. gracilipes* abundance and genetic diversity measures was not significant for ^0^*D* (*S* = 674, *P* = 0.109, *R*_s_ = 0.30), this measure of diversity nonetheless had a medium sized positive correlation with abundance. These relationships were positive and significant for ^1^*D* (*S* = 431, *P* = 0.008, *R*_s_ = 0.55), ^2^*D* (*S* = 1514, *P* = 0.007, *R*_s_ = 0.56), and genotypic diversity (*S* = 540, *P* = 0.034, *R*_s_ = 0.44). All significant relationships also had large effect sizes.

**Table 1:** Abundance and genetic diversity parameters for *Anoplolepis gracilipes* for the 18 invaded plots in the study

			Allelic richness				Genetic diversity			Genotypic richness	
					
Plot	Abundance	*N*	*Ano3*	*Ano7*	*Ano8*	Other loci	^0^*D*	^1^*D*	^2^*D*	*G*	*R*_U_
A1	1353	31	2.00	2.00	6.43	2.00	10.43	4.16	1.48	7	0.20
A2	452	26	2.00	2.00	4.74	2.00	8.74	3.23	1.45	4	0.12
B1	5288	23	2.99	2.85	8.86	2.00	14.58	11.72	1.07	14	0.59
B2	898	25	2.87	4.60	10.01	2.00	16.61	13.59	1.05	16	0.63
D1	139	33	3.47	3.57	4.22	2.00	11.26	5.82	1.25	9	0.25
D2	1577	32	3.00	3.61	4.26	2.00	10.87	5.85	1.25	9	0.26
D3	1375	21	2.00	2.00	7.00	2.00	11.00	8.30	1.12	10	0.45
D4	3357	30	2.67	2.00	7.54	2.00	12.21	9.95	1.09	12	0.38
G1	2320	35	2.00	2.79	6.77	2.00	11.56	7.80	1.14	10	0.27
G2	432	22	2.00	2.92	6.91	2.00	11.83	5.72	1.21	7	0.29
K2	566	20	2.00	2.50	9.00	2.00	13.00	6.68	1.18	9	0.42
K4	1183	25	2.79	3.00	8.54	2.00	14.33	9.98	1.09	12	0.46
K6	353	35	2.99	3.00	7.81	2.00	13.80	8.37	1.14	11	0.29
K7	256	27	2.00	2.93	6.69	2.00	11.62	5.82	1.24	9	0.31
P1	350	30	2.00	2.70	5.34	2.00	10.04	3.32	1.65	6	0.17
P2	383	29	2.00	2.69	4.60	2.00	9.29	2.42	2.18	5	0.14
M1	62	26	2.00	3.96	4.67	2.00	10.63	4.40	1.38	7	0.24
M2	76	28	2.00	3.92	5.35	2.00	11.27	4.70	1.43	8	0.26

Abundance, the total number of ants caught in pitfall traps; *N*, number of workers genotyped; Allelic richness, rarefied allelic richness for each locus based on the smallest sample size (21); ^0^*D*, rarefied allelic richness for the three informative loci; ^1^*D*, exp[Shannon's *H′*]); ^*2*^*D*, (1/Simpson's index *D*); *G*, multi-locus genotypes (genotypic richness); *R*_U_, unbiased genotypic diversity.

### Are there differences in species diversity and community structure between invaded and uninvaded communities?

Seventy species in total were found in our survey (61 in uninvaded and 49 in invaded plots), and species richness per plot ranged from 7 to 22 species (mean ± SE: 12 ± 0.6 species in invaded plots and 15 ± 0.8 species in uninvaded plots). Mean native ant abundances ranged from 52 to 918 ants per plot (mean ± SE: 252 ± 42 ants in invaded and 319 ± 57 ants in uninvaded plots).

**Figure 4:**
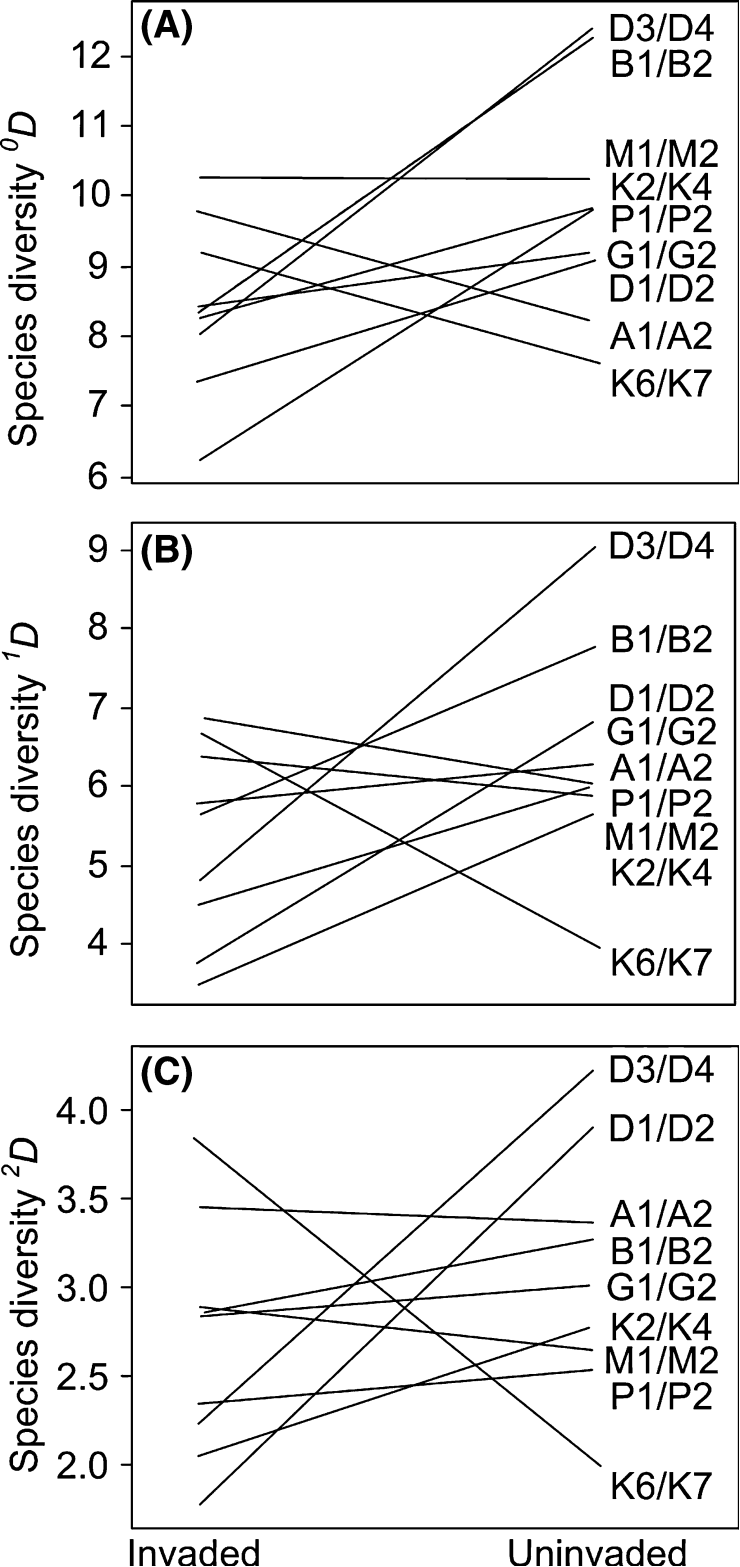
Interaction plots of ant species diversity differences between *Anoplolepis gracilipes* invaded and uninvaded plots (grouped by site) for: (A) ^0^*D* (species richness); (B) ^1^*D* (exp[Shannon's *H'*]); and (C) ^2^*D* (1/Simpson's index *D*). The letters on the right side of the interaction plots represent the sites shown in Fig. 2.

Only species richness (^0^*D*) was significantly higher in uninvaded plots than invaded plots (Fig. 4; approximative Wilcoxon Mann–Whitney Rank Sum Test, *Z* = −1.96, *R*^2^ = 0.11, *P* = 0.047). Other species diversity measures did not significantly differ between invaded and uninvaded plots, but the observed effect sizes were progressively lower as *q* increased. (^1^*D*: *Z* = −1.76, *R*^2^ = 0.09, *P* = 0.077; ^2^*D*: *Z* = −1.24, *R*^2^ = 0.04, *P* = 0.227). As rare species carry a lower importance in the ^1^*D* and ^2^*D* measures, this decreasing effect size suggests that rare species are less likely to co-occur with *A. gracilipes*.

**Figure 5:**
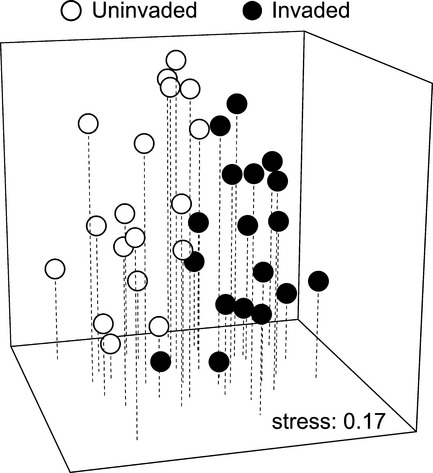
Three-dimensional MDS plots of *Anoplolepis gracilipes* invaded and uninvaded plots based on Bray-Curtis similarity of log (*X* + 1) transformed abundance data. *Anoplolepis gracilipes* were excluded from the analysis. The accompanying ANOSIM analysis revealed significant differences between sites (Global *R* = 0.33, *R*^2^ = 0.11, *P* = 0.040), and between invaded and uninvaded sites (Global *R* = 0.44, *R*^2^ = 0.19, *P* = 0.010).

Three-dimensional MDS revealed clear differences in community structure between uninvaded and invaded plots (Fig. 5). The results of ANOSIM analysis revealed significant differences between sites (Global *R* = 0.33, *R*^2^ = 0.11, *P* = 0.040) and between treatments (Global *R* = 0.44, *R*^2^ = 0.19, *P* = 0.010). The significance of site effects was consistent with our results for species diversity measures (Fig. 4) where species diversity was not always lower in uninvaded than invaded plots. Thus, differences between sites as well as invasion status contributed to differences in community structure.

SIMPER analysis revealed that 90% of the dissimilarity between invaded and uninvaded sites was accounted for by 33 ant species, whereas 50% of the dissimilarity was accounted for by 11 species. The abundances of a number of these species differed markedly between invaded and uninvaded plots (Table 2). The contribution of different functional groups also differed between invaded and uninvaded plots. Invaded plots had fewer Dominant Dolichoderinae (DD) and Tropical Climate Specialists (TCS) and more Generalized Myrmicinae (GM). Ecologically dominant species (DD and *Oecophylla smaragdina*) were absent from many but not all invaded plots. In invaded sites, *O. smaragdina* occurred only in three plots (M1, M2 and P2). Only two species of DD occurred at invaded sites. Of these, *Iridomyrmex pallidus* was found at plots B2, D3, D4, G2, K2, and K4. *Iridomyrmex* sp. 1 *anceps* group was found at plots G2 and P2. When they occurred in invaded plots these species had lower abundance than when they were found in uninvaded plots (Table 2), whereas other *Iridomyrmex* species were only found in uninvaded plots.

**Table 2:** Differences in the abundances (log-transformed) of the ant species and functional groups that contributed to ∼90% of the dissimilarity between *Anoplolepis gracilipes* invaded sites compared with uninvaded sites in northeast Arnhem Land, Australia. *Anoplolepis gracilipes* was excluded from the analyses

	Mean abundance		%		
			
	Uninvaded	Invaded	Difference	Contribution	Cumulative % contribution
Species					
*Pheidole* sp. 3 *variabilis* group	0.61	2.12	247.5	5.5	
*Pheidole* sp. 8 group F	1.24	2.05	65.3	5.3	10.8
*Nylanderia* sp. 4 *vaga* group	2.17	0.39	−82.0	5.2	16.0
*Monomorium* sp. 8 *carinatum* group	2.25	1.15	−48.9	5.2	21.2
*Monomorium* sp. A *nigrius* group	1.99	1.07	−46.2	4.9	26.1
*Nylanderia* sp. 13 *vaga* group	0.65	1.86	186.2	4.8	30.9
*Crematogaster queenslandica*	0.92	1.47	59.8	4.0	34.9
*Oecophylla smaragdina*	1.67	0.25	−85.0	4.0	38.9
*Monomorium* sp. 46 *laeve* group	1.14	1.18	3.5	3.6	42.5
*Tetramorium* sp. 1 *striolatum* group	1.40	1.59	13.6	3.6	46.1
*Monomorium* sp. 24 *laeve* group	3.18	3.46	8.8	3.3	49.4
*Iridomyrmex* sp. 1 *anceps* group	1.09	0.60	−45.0	3.3	52.7
*Odontomachus* sp. near *turneri*	1.07	0.14	−86.9	2.8	55.5
*Iridomyrmex pallidus*	1.11	0.39	−64.9	2.8	58.3
*Camponotus* sp. 11	1.14	0.61	−46.5	2.7	61.0
*Paraparatrechina* sp. 2 *minutula* group	0.88	0.78	−11.4	2.7	63.7
*Pheidole impressiceps*	0.27	0.63	133.3	2.2	65.9
*Pheidole* sp. A *variabilis* group	0.37	0.55	48.6	2.1	68.0
*Monomorium* sp. 13 *nigrius* group	0.88	0.15	−83.0	2.1	70.1
*Opisthopsis haddoni*	0.73	0.53	−27.4	2.0	72.1
*Meranoplus* sp. 8 group F	0.08	0.70	775.0	2.0	74.1
*Tetramorium lanuginosum*	0.34	0.44	29.4	1.8	75.9
*Iridomyrmex reburrus*	0.73	0.00	−100.0	1.8	77.7
*Rhytidoponera* sp. 9 *tenuis* group	0.44	0.49	11.4	1.8	79.5
*Meranoplus mjobergi*	0.43	0.41	−4.7	1.7	81.2
*Tetramorium simillimum*	0.10	0.58	480.0	1.6	82.8
*Crematogaster* sp. 2 *laeviceps* group	0.56	0.06	−89.3	1.4	84.2
*Solenopsis* sp. 1	0.66	0.04	−93.9	1.4	85.6
*Rhytidoponera* sp. 3 *turneri* group	0.22	0.23	4.5	1.1	86.7
*Iridomyrmex* sp. 3 *mjobergi* group	0.30	0.06	−80.0	0.9	87.6
*Rhytidoponera aurata*	0.34	0.00	−100.0	0.9	88.5
*Tapinoma* sp. 1	0.15	0.22	46.7	0.8	89.3
*Polyrhachis inconspicua*	0.22	0.22	0.0	0.8	90.1
Functional group					
*Dominant Dolichoderinae (DD)*	2.61	0.84	−67.8	23.0	
*Generalized Myrmicinae (GM)*	3.12	4.09	31.1	18.0	41.0
*Tropical Climate Specialists (TCS)*	1.67	0.28	−83.2	16.7	57.7
*Hot Climate Specialists (HCS)*	4.45	4.02	−9.7	11.9	69.6
*Opportunists (OPP)*	3.63	3.46	−4.7	11.46	81.1
*Subordinate Camponotini (SC)*	1.82	1.32	−27.5	10.77	91.9

### Is there an association between *Anoplolepis gracilipes* abundance and species diversity of the invaded community?

**Figure 6:**
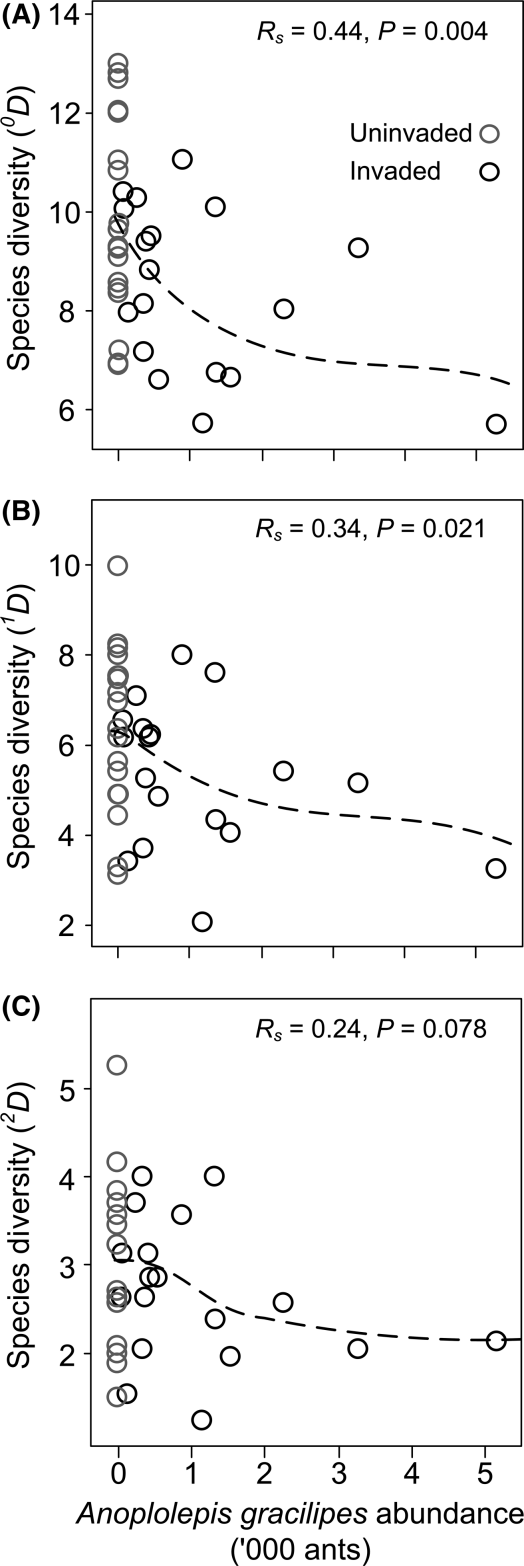
Relationships between *Anoplolepis gracilipes* abundance and native ant: (A) ^0^*D* (species richness); (B) ^1^*D* (exp[Shannon's *H′*]); and (C) ^2^*D* (1/Simpson's index *D*). Dashed lines indicate the smoothed spline line of best fit with a span of 0.9.

Greater *A. gracilipes* abundance was associated with lower native ant species diversity for all measures of species diversity (Fig. 6), but the effect size and statistical significance of this relationship was lower for the measures that placed less importance on rare species (^0^*D*: *S* = 11151, *R*_s_ = 0.44, *P* = 0.004; ^1^*D*: *S* = 10422, *R*_s_ = 0.34, *P* = 0.021; ^2^*D*: *S* = 9647, *R*_s_ = 0.24, *P* = 0.078). This result mirrored our finding that species diversity did not differ between invaded and uninvaded plots when rare species were assigned less importance, and supports our finding that rare species are less likely to co-occur with *A. gracilipes*. The relationships between abundance and species diversity were not linear, and instead were best fit by lines that tended toward a sigmoidal function.

### Are habitat characteristics associated with differences in *Anoplolepis gracilipes* abundance?

None of the habitat characteristics we measured were significantly correlated with differences in *A. gracilipes* abundance. For all plots combined the best model included site as a fixed factor and the canopy, *Acacia*, and litter variables. The best model explained 48% of the variation in the data, but no variables were statistically significant (*R*^2^ = 0.20, canopy: *F* = 1.02, *P* = 0.324, *Acacia*: *F* = 1.38, *P* = 0.273, and litter: *F* = 3.06, *P* = 0.080). For invaded plots only the best statistical model included site and *Acacia*, and explained 65% of the variation in the data, but again, no variables were statistically significant (*R*^2^ = 0.18, *Acacia*: *F* = 0.94, *P* = 0.44). The PCA also did not reveal any differences between invaded and uninvaded plots among the measured habitat characteristics (Supplementary Fig. S3).

## Discussion

### Correlations between genetic diversity and abundance

We found that higher abundance of *A. gracilipes* was significantly correlated with higher genetic diversity, which is consistent with the evolutionary theoretical expectations that populations with higher genetic diversity should experience greater invasion success (Sakai et al. 2001). The apparent genetic paradox of invasion, in which invading species are successful despite population bottlenecks that should limit invasion success (Allendorf and Lundquist 2003), is not reflected in our results. Although allelic richness (^0^*D*) was not significantly correlated with higher abundance of *A. gracilipes* in Arnhem Land, the relationship between the two was positive and moderately large. Genotypic diversity and ^1^*D* diversity were significantly positively correlated with higher *A. gracilipes* abundance, but ^2^*D* diversity was negatively correlated with higher abundance. The remainder of our discussion focuses on genotypic diversity because: (1) it relates directly to individuals; (2) may indicate whether multiple queens or males contribute to reproduction; and (3) may be interpreted in light of asexual reproduction, which has been suggested possibly contributes to the reproductive mode of *A. gracilipes* (Drescher et al. 2007; Heinze 2008).

Nest clusters of *A. gracilipes* could benefit from higher genetic diversity in a number of ways. Whereas higher genetic diversity may not increase short-term task efficiency (Rosset et al. 2005), a number of studies have found that higher genetic diversity among social insect workers offers a range of positive benefits. These benefits include reduced parasitic infection (Sherman et al. 1988; Shykoff and Schmid-Hempel 1991; Keller 1995; Tarpy 2003), enhanced colony growth (Cole and Wiernasz 1999; Tarpy 2003), and productivity and fitness (Mattila and Seeley 2007). Although it remains to be seen whether the fine-scale, momentary correlation between genetic diversity and abundance that we observed translates into invasion success at larger spatial and temporal scales, higher genetic diversity of the worker population may have positive effects at the local scale.

How then might variation in genetic diversity between nest clusters affect the dynamics of the greater *A. gracilipes* population in Arnhem Land? *Anoplolepis gracilipes* populations have been known to vary in abundance temporally, significantly decline, or collapse entirely (e.g., Haines and Haines 1978b; Abbott 2006; B. Hoffmann, personal observation). If genetic diversity drives abundance, meta-population dynamics might result in “sink” populations (Pulliam 1988) of lower genetic diversity that do not persist. The fluid nature of the population structure of *A. gracilipes* in Arnhem Land, and lack of significant aggression between geographically distant nests (Gruber et al. 2012) also suggests that “sink” nest clusters may be able to receive additional propagules from “source” nest clusters, depending on their degree of geographical isolation. If the relationship is reversed, and abundance drives higher genetic diversity, larger propagules may have better chances of persistence. Longer term study of the correlations between abundance and genetic diversity, and the spatio-temporal dynamics of abundance in *A. gracilipes* would reveal if this was the case in Arnhem Land.

Although our findings appear to be in contrast to the hypothesis that genetic bottlenecks may promote invasion success, which has been suggested for *L. humile* (Suarez et al. 1999; Tsutsui et al. 2000), potential positive effects of a bottleneck for *A. gracilipes* may only occur at the introduction event. As the invasion of *A. gracilipes* in Arnhem Land likely stemmed from a single source population that has since diverged (Gruber et al. 2012), any effects of the original population genetic bottleneck are no longer observable. Ideally, to determine the effects of potential bottlenecks that may affect the success of *A. gracilipes* in the invaded range we would need a comparison with the native range, and longer term studies with repeated sampling. Unfortunately the native range of *A. gracilipes* is unknown, although the ant is suspected to have Asian origins (Wetterer 2005; Drescher 2011; Sébastien et al. 2012). Thus, whether the original bottleneck promoted establishment success in the Arnhem Land population is unknown, but may be possible.

Variation in genetic diversity may be responsible for variation in the abundance of *A. gracilipes* in Arnhem Land, or more abundant populations may be more genetically diverse because more individuals contribute to reproduction. In ant societies, census population size (*N*_c_ – workers together with reproductives) and effective population size (*N*_e_ *–* individuals contributing to reproduction) are potentially decoupled, as reproduction often involves few individuals, and these are not workers (Wilson 1963). We assumed this might be the case for *A. gracilipes*. However, our results show that genotypic diversity and abundance are coupled. In addition, there is the suggestion that *A. gracilipes* workers may contribute to reproduction (Heinze 2008), in which case worker abundance and genetic diversity could be more closely coupled if more worker clones result in higher abundance.

Ant colonies can also be more genetically diverse if more queens and/or males contribute to reproduction (i.e., multiple queens reproduce [polygyny], or queens mate with multiple males [polyandry]: Pamilo 1991). Although the number of queens in *A. gracilipes* nests in Arnhem Land varies (up to 16 queens per nest: M. Gruber, B Hoffmann and P. Lester, unpubl. data) and is often much higher (up to 300 queens per nest in the Seychelles: Haines and Haines 1978a), polygyny has not been investigated in the species. If polygyny contributes significantly, a decline in queen abundance may result in lower genetic diversity within the nest (or nest cluster). Clearly, further exploration of the dynamics of colony structure and the reproductive mode of *A. gracilipes* are required to answer these questions.

### Community structure and diversity differ between invaded and uninvaded sites

The effect of invading ants on the recipient ant community does not always result in a decline in species richness, as some species may increase in abundance while others decrease (Guénard and Dunn 2010). We used a combination of diversity measures, which revealed more regarding the nature of differences between communities than species richness alone. Species richness was not always lower in invaded plots than uninvaded plots. While rare species were less likely to occur with *A. gracilipes*, small, inconspicuous, insinuating species were commonly present. Other studies of *A. gracilipes* have found lower ant species richness in invaded sites (e.g., Sarty et al. 2007; Savage et al. 2009; Drescher et al. 2011), and that ecologically dominant species are less likely to co-occur with *A. gracilipes* (Hoffmann and Saul 2010). When *A. gracilipes* reaches high abundance it causes more marked changes to ant community structure (Abbott et al. 2007; Lester et al. 2009). So while the absence of rare ant species in the presence of *A. gracilipes* may be of conservation concern, the patchy distribution and variable abundance of *A. gracilipes* in Arnhem Land is unlikely to have significant effects on regional native species diversity.

### Habitat characteristics may be less important than genetic diversity

When viewed in a biogeographical context it is clear that many factors influence invasion success (Wilson et al. 2009). Although many introduced populations experience a reduction in genetic diversity in the invaded range (e.g., Grapputo et al. 2005; Zayed et al. 2007; Dlugosch and Parker 2008), they may thrive if the new ecological conditions are more favorable than the native range (Sax and Brown 2000; Colautti et al. 2004; Moles et al. 2008). Alternatively, higher genetic diversity may be a more important contributor to invasion success where novel ecological conditions do not promote abundance. We suggest that this may be the case for *A. gracilipes* in Arnhem Land, where we found no association between the habitat characteristics we assessed and *A. gracilipes* abundance, yet there was clearly a significant positive effect of higher genetic diversity on abundance.

Of the habitat variables we measured, carbohydrate resources are perhaps the most important driver of *A. gracilipes* abundance elsewhere (O'Dowd et al. 1999, 2003; Savage et al. 2009), and strongly influence ant abundance generally (Davidson 1997 and references therein). Thus, it was surprising that we found no effect of the presence of Acacia on the abundance of *A. gracilipes*. The abundance of *A. gracilipes* in Arnhem Land appears very low compared with the ant on Christmas Island (our card counts ranged from 0 to 43, compared with a range of ∼14–136 on Christmas Island, Abbott 2005), where *A. gracilipes* abundance is facilitated by honeydew-exuding scale insects (O'Dowd et al. 2003). The lesser abundance we observed may be due to a lack of exploitation or availability of carbohydrate resources in Arnhem Land. Conversely, *A. gracilipes* may not have reached (or be able to reach) a minimum level of abundance required to monopolize exudate producing resources, as found in *Technomyrmex albipes* (Oliver et al. 2008). Regardless of the direction of the relationship between carbohydrate resources and abundance, our results suggest that in the absence of clear ecological drivers of abundance, genetic diversity may be a more important factor in invasion success. We do not deny that habitat characteristics, and particularly novel resources, influence invasion success (and ecological dominance generally), but in this case the effect of genetic diversity was much stronger than the habitat variables we measured.

## Conclusions

We found evidence of a positive association between genetic diversity and abundance in *A. gracilipes* in Arnhem Land. Although higher genetic diversity may benefit individual nest clusters, the underlying mechanisms and the direction of the relationship between abundance and genetic diversity are unclear, and the implications for longer term invasion success on the wider population are difficult to predict. Although our results are in contrast to the hypothesis that genetic bottlenecks may promote unicoloniality in *L. humile* (Tsutsui et al. 2000), the population divergence of *A. gracilipes* subsequent to introduction in Arnhem Land has obscured evidence of the bottleneck that would likely have occurred on arrival. The relative importance of genetic diversity to the success of founding populations may be context-dependent, may change over time, and be more obvious in the absence of highly favorable ecological characteristics.

## Supporting Information

**Figure S1**. Allele Discovery curves for the most polymorphic locus (*Ano8*) for all 18 plots (grouped by site) in the study. Where curves have not flattened completely there were no further samples for genotyping.

**Figure S2**. Species accumulation curves for: (A) invaded and (B) uninvaded plots (grouped by site) in the study.

**Figure S3.** Principal component analysis of *Anoplolepis gracilipes* invaded and uninvaded sites based on Euclidean distance of standardized values for availability of nest sites, canopy cover, presence of *Acacia* spp., and leaf litter depth.
