# Supplementary material for: Genetic diversity is positively associated with fine-scale momentary abundance of an invasive ant
Source: Ecol Evol. 2012 Jul 24;2(9):2091–105. doi: 10.1002/ece3.313 (PMC3488662; doi:10.1002/ece3.313)

# Appendix

Supplementary Fig. S1: Allele Discovery curves for the most polymorphic locus (*Ano8*) for all 18 plots (grouped by site) in the study. Where curves have not flattened completely there were no further samples for genotyping.


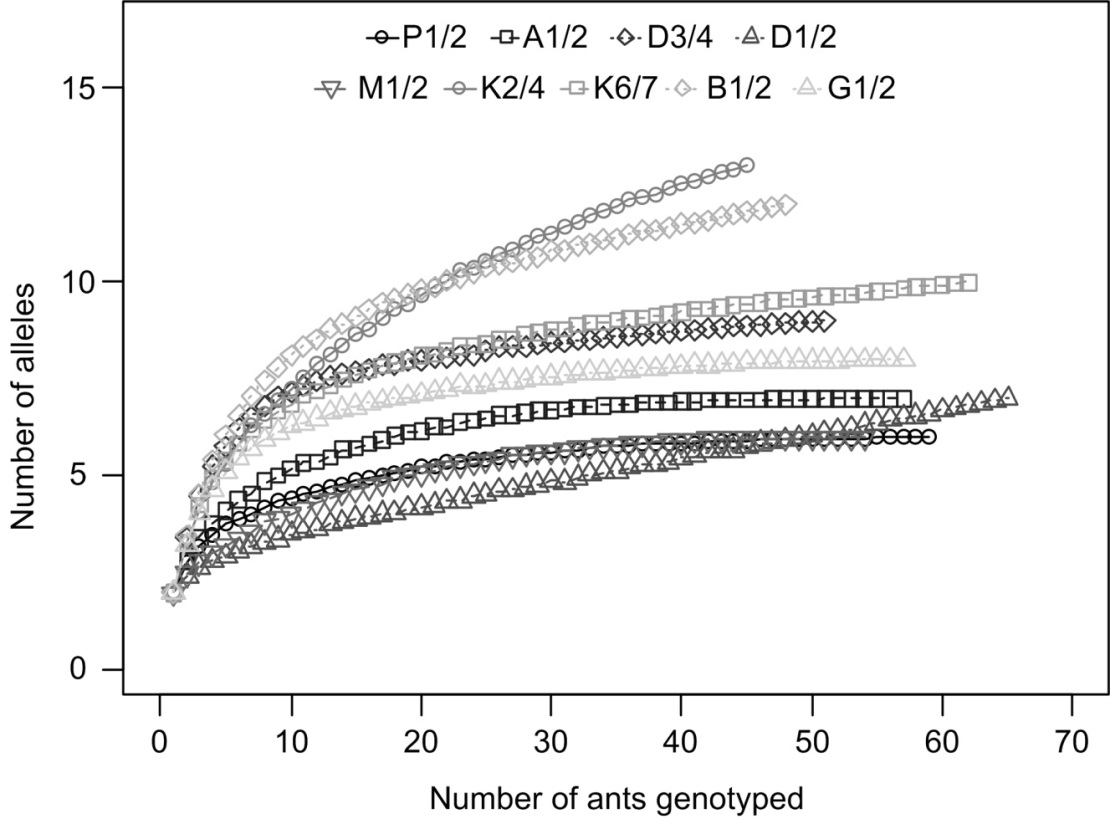


Supplementary Fig. S2: Species accumulation curves for: A) invaded; and B) uninvaded plots (grouped by site) in the study.

Supplementary Fig. S3: Principal component analysis of *Anoplolepis gracilipes* invaded and uninvaded sites based on Euclidean distance of standardised values for availability of nest sites, canopy cover, presence of *Acacia* spp. and leaf litter depth.


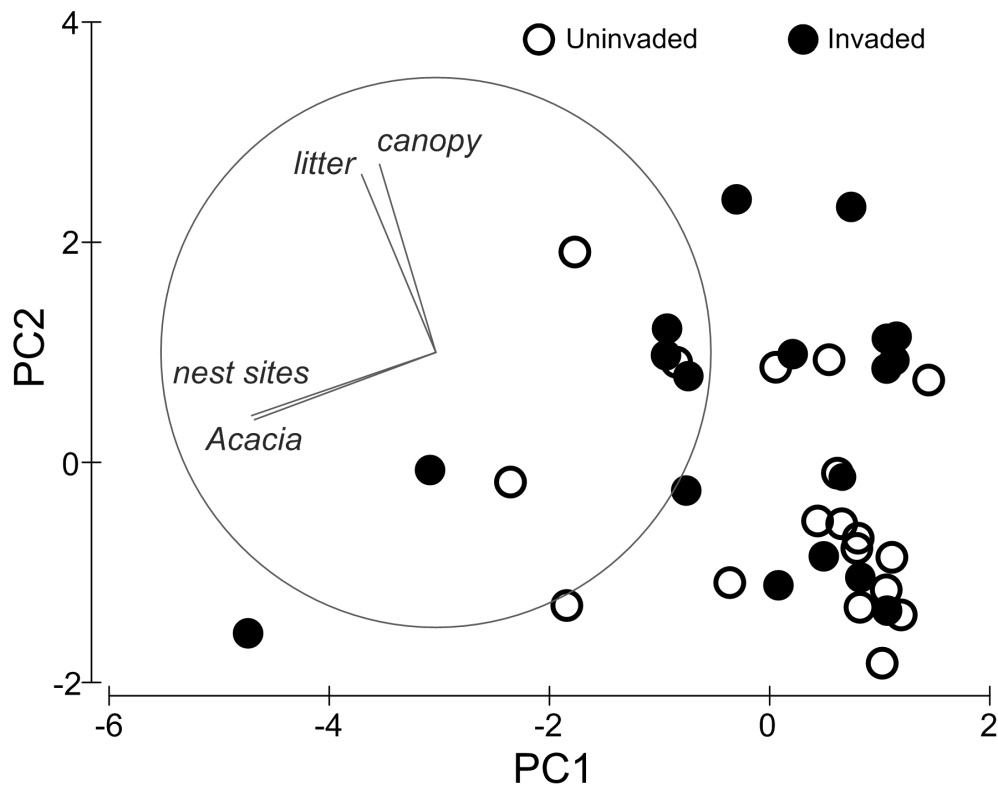

Supplement: Supplementary file 1 [file ece30002-2091-SD1.docx]
